# Integrating molecular targeting and immune modulation in triple-negative breast cancer: from mechanistic insights to therapeutic innovation

**DOI:** 10.3389/fimmu.2025.1711415

**Published:** 2025-11-26

**Authors:** Yueren Fan, He Wang, Hongyu Zhang, Tianfei Ma, Yihang Zhao

**Affiliations:** Department of Breast Oncology II, Cancer Hospital of Dalian University of Technology (Liaoning Cancer Hospital), Shenyang, Liaoning, China

**Keywords:** triple-negative breast cancer, tumor-infiltrating lymphocytes, antibody-drug conjugates, immunotherapy, antitumor immunity

## Abstract

Triple-negative breast cancer (TNBC) remains a clinically aggressive subtype of breast cancer, defined by the absence of estrogen receptor, progesterone receptor, and HER2 amplification, and disproportionately affecting younger and racially diverse populations. Despite conventional chemotherapy, TNBC patients often face poor prognoses due to the lack of actionable molecular targets and early metastatic potential. Advances in molecular profiling have unveiled distinct TNBC subtypes and actionable vulnerabilities, including BRCA1/2 mutations and PI3K/AKT/mTOR dysregulation. Therapies targeting DNA repair pathways, angiogenesis, and androgen receptor signaling—particularly via PARP inhibitors and antibody–drug conjugates like sacituzumab govitecan—have demonstrated clinical benefit. Concurrently, TNBC’s immunogenic nature, reflected in dense tumor-infiltrating lymphocytes (TILs), has driven the integration of immune checkpoint inhibitors. However, both primary and acquired resistance remain major barriers. This review delineates recent developments in targeted and immunotherapeutic strategies, emphasizing the role of TILs in shaping treatment response and highlighting combinatorial approaches that synergize molecular targeting with immunomodulation. Through a comprehensive understanding of TNBC’s molecular and immune landscape, we propose new therapeutic trajectories to improve clinical outcomes in this challenging malignancy.

## 1 Introduction

Breast cancer persists as the most frequently diagnosed malignancy among women on a global scale, with incidence rates demonstrating a consistent upward trend in recent years (1). Among its subtypes, triple-negative breast cancer (TNBC) accounts for approximately 15% of cases and is marked by unique epidemiological features, showing higher prevalence in younger, premenopausal women and specific racial minorities (1). The absence of actionable molecular targets, such as hormone receptors and HER2 amplification, renders endocrine therapy and anti-HER2 strategies largely ineffective (2, 3). Even when aggressive multimodal treatment is employed, patients with advanced TNBC face significantly worse survival outcomes compared to those with other breast cancer subtypes (4, 5). The current therapeutic impasse underscores the urgent demand for innovative interventions capable of improving prognosis in this biologically aggressive disease.

Comprehensive molecular profiling has uncovered the intrinsic heterogeneity of TNBC, leading to its subclassification into distinct molecular entities, including basal-like, mesenchymal-like, immunomodulatory, androgen receptor-positive, and HER2-enriched phenotypes, each governed by separate signaling mechanisms (6–8). Notably, TNBC exhibits heightened immunogenicity, as evidenced by the dense infiltration of tumor-infiltrating lymphocytes (TILs), providing a biological basis for the application of immune checkpoint inhibitors. Multiple clinical studies have demonstrated promising therapeutic responses to such immunomodulatory strategies (1). This review synthesizes recent advancements in molecularly tailored treatments and TIL-focused immunotherapies, with particular emphasis on their translational value and potential trajectories for future clinical development in TNBC.

## 2 Advances in molecularly oriented therapeutics

### 2.1 Intervening in DNA repair pathways and growth factor cascades

A subset of TNBC, approximately 15%, carry inherited mutations in the BRCA1 or BRCA2 genes, which disrupt the homologous recombination (HR) pathway and render tumor cells especially susceptible to agents inducing DNA damage (9). This molecular vulnerability has been clinically harnessed through the application of platinum-based drugs and poly (ADP-ribose) polymerase (PARP) inhibitors, which have demonstrated significant efficacy in BRCA-mutated TNBC populations (10, 11). The Phase II trial broadened the therapeutic scope by administering olaparib to patients with advanced TNBC characterized by homologous recombination deficiency (HRD) yet lacking germline BRCA1/2 mutations. The trial reported a clinical response rate of 50%, thereby supporting the extension of PARP inhibitor use to HRD-positive, BRCA-wild-type individuals (12). Beyond their genotoxic effects, PARP inhibitors contribute to immunogenic tumor remodeling. The accumulation of DNA damage promotes the release of neoantigens, thereby enhancing antigen uptake and presentation by dendritic cells, which subsequently activate T cells (13, 14). This cascade primes adaptive immune responses, increasing the cytotoxic potential of effector lymphocytes. Concurrently, the presence of unrepaired DNA fragments in the cytoplasm activates the cGAS–STING signaling axis, which functions as a sentinel for genomic perturbation (15). Under persistent genotoxic stress, PARP1 localizes to DNA lesions, facilitating cyclic GMP–AMP production by cGAS. This second messenger binds to and activates STING, leading to phosphorylation cascades involving IRF3 and NF-κB (16). As a result, type I interferons (notably IFN-β) and pro-inflammatory mediators such as TNF-α and IL-6 are transcriptionally upregulated, bolstering immune surveillance and anti-tumor responses (17–19). Simultaneously, PARP inhibition increases MHC class I surface expression on malignant cells (20), thereby improving recognition by cytotoxic CD8^+^ T lymphocytes (21–24). IFN-γ further amplifies this antigen-presenting capacity and stimulates effector immune cell infiltration within the tumor microenvironment (25, 26). DNA damage response pathways, including ATM and ATR signaling, are also activated and propagate downstream via CHK1 and CHK2, orchestrating cell cycle arrest and DNA repair mechanisms (27–29). These pathways not only maintain genomic integrity but also modulate the immune landscape by influencing leukocyte recruitment and functionality, thus enhancing conditions conducive to T cell–mediated cytotoxicity (30). Furthermore, PARP-induced DNA lesions may drive phenotypic shifts in tumor-associated macrophages, promoting a transition from the immunosuppressive M2 phenotype toward the pro-inflammatory M1 subtype. This macrophage repolarization serves to reinforce anti-tumor immunity and contributes to tumor regression (31). Notably, increasing evidence supports the synergistic antitumor efficacy of combining PARP inhibitors with ICIs in TNBC. Preclinical models have shown that PARP inhibition enhances immune checkpoint blockade (ICB) efficacy by promoting neoantigen release, activating the cGAS–STING–IFN axis, and upregulating PD-L1 expression, thereby creating a more immunostimulatory tumor microenvironment (32, 33). In murine TNBC models, the combination of olaparib with anti–PD-L1 therapy led to improved tumor regression and increased infiltration of functional CD8^+^ T cells compared to either agent alone (34). These findings validate the mechanistic rationale and provide a clinical framework for the integration of PARP–ICI combinations in biomarker-defined TNBC subsets.

### 2.2 Angiogenesis and proliferative signaling pathways

TNBC is frequently characterized by pronounced overexpression of vascular endothelial growth factor (VEGF), a key driver of pathological neovascularization within tumors (35). Anti-angiogenic therapies, however, have shown only limited clinical success in this context (36, 37). A phase III clinical trial demonstrated that combining bevacizumab, a monoclonal antibody targeting VEGF, with standard chemotherapy resulted in a significant extension of progression-free survival compared to chemotherapy alone in patients with advanced TNBC, although this did not translate into a statistically significant improvement in overall survival (38). However, the limited survival benefit of anti-VEGF therapy in TNBC can be attributed to several factors. First, tumor angiogenesis is often mediated by multiple redundant pathways beyond VEGF alone, such as fibroblast growth factor (FGF) and angiopoietin signaling, which may compensate upon VEGF blockade (39). Second, adaptive resistance mechanisms—such as increased pericyte coverage, vessel co-option, and hypoxia-induced pro-invasive gene expression—may enable tumors to circumvent the anti-angiogenic effects (40). Third, the inherently immunosuppressive tumor microenvironment in TNBC, exacerbated by abnormal vasculature, can impair immune cell infiltration and blunt the potential synergy between anti-angiogenic and immunotherapeutic agents (41). These limitations highlight the need for rational combination strategies and biomarker-guided patient selection to optimize clinical outcomes. Despite this drawback, the therapeutic combination continues to be administered in select regions, where certain patient subpopulations derive measurable benefit (42–44). In addition to antibody-based interventions, broad-spectrum tyrosine kinase inhibitors such as sunitinib—which concurrently inhibit VEGF and platelet-derived growth factor (PDGF) receptors—have shown efficacy in preclinical models by disrupting angiogenic signaling and reducing tumor mass (45). These agents are designed to remodel disorganized vasculature, thereby enhancing oxygen delivery and improving the penetration of co-administered treatments (46). Aberrant activation of the PI3K/AKT/mTOR signaling cascade represents another critical feature of TNBC, sustaining proliferative advantage, metabolic shifts, and resistance to programmed cell death (47, 48). Targeted blockade of this axis has yielded encouraging results; for instance, the mTOR inhibitor everolimus was found to suppress proliferation in basal-like TNBC cell cultures, and early-phase clinical investigations involving its combination with cytotoxic agents have achieved disease control in specific patient cohorts (49). Furthermore, advanced-generation inhibitors of PI3K and AKT are undergoing clinical trials, with the objective of enhancing therapeutic index while limiting adverse effects (50–53). Nonetheless, due to the fundamental involvement of this signaling network in normal cellular function, careful stratification of patients and vigilant toxicity monitoring are indispensable for clinical translation.

### 2.3 Antibody–drug conjugates and androgen receptor

Antibody–drug conjugates (ADCs) represent a transformative class of therapeutics, coupling monoclonal antibody specificity with potent cytotoxic payloads. In TNBC, Trop-2 is overexpressed in >90% of tumors, rendering it a compelling target. Sacituzumab govitecan, comprising an anti–Trop-2 antibody linked to SN-38 (the active metabolite of irinotecan), induces DNA double-strand breaks and apoptosis in Trop-2^+^ cells (54). Phase II (IMMU-132-01) and phase III (ASCENT) trials demonstrated substantial efficacy and survival benefits in heavily pretreated metastatic TNBC cohorts (55). A distinct TNBC subset expresses androgen receptor (AR), enabling endocrine-targeted interventions (56). Enzalutamide, a first-generation AR antagonist, achieved a 35% clinical benefit rate in metastatic TNBC (57, 58). However, transient efficacy has prompted investigation of next-generation AR inhibitors (darolutamide, apalutamide), which exhibit superior preclinical activity and may synergize with chemotherapy or immune checkpoint inhibitors to suppress AR-driven signaling and overcome resistance (59–61). Preclinical studies suggest that AR inhibition can reprogram the immunosuppressive tumor microenvironment, enhancing T cell cytotoxicity and potentiating ICI efficacy (62). Mechanistically, tumor-derived exosomal miR-205-3p downregulates AR, promoting epithelial–mesenchymal transition (EMT) and metastatic spread (63–65), while C/EBPβ-mediated activation of kinesin family member C1 (KIFC1) drives EMT and invasiveness in AR^+^ TNBC (64, 66). Bidirectional crosstalk between AR and epidermal growth factor receptor (EGFR) pathways further implicates therapeutic synergy, suggesting that androgen deprivation therapy (ADT), established in prostate cancer, may offer utility in AR^+^ TNBC either concurrently or sequentially (67–69). Collectively, these findings emphasize the clinical promise of AR-targeted interventions, especially when rationally combined with established or emerging agents, in refining therapeutic regimens for TNBC subtypes characterized by AR expression.

## 3 TILs and their clinical significance in TNBC

### 3.1 Immune cell landscape and evaluation strategies

TNBC is distinguished by a tumor microenvironment (TME) rich in infiltrating immune cells, collectively known as TILs (70, 71). These lymphocyte populations—comprising primarily T cells, along with B lymphocytes and natural killer (NK) cells—are located both within the malignant epithelial compartments and adjacent stromal zones, serving as key indicators of endogenous antitumor immune surveillance (72, 73). Compared to other breast cancer subtypes, TNBC exhibits the most substantial immune cell infiltration, reinforcing its classification as the most immunogenic form of breast malignancy (74). Clinically, an increased density of TILs has been associated with superior responses to chemotherapy and improved long-term outcomes. This relationship is particularly evident in the neoadjuvant setting, where elevated baseline TIL levels reliably forecast a higher likelihood of achieving pathological complete response (pCR) and extended survival (75, 76). Within this immunological context, T cells represent the dominant fraction, with CD8^+^ cytotoxic and CD4^+^ helper subsets comprising the majority of the lymphocytic infiltrate. CD8^+^ cytotoxic T lymphocytes (CTLs) represent the principal effector population mediating direct tumor cell lysis and are strongly associated with improved prognosis in TNBC (77). Conversely, CD4^+^ T helper cells exhibit context-dependent roles—Th1 cells secrete IFN-γ and bolster antitumor immunity, while Th2 and Th17 subsets may contribute to tumor inflammation and immune escape (78). Although less abundant, B lymphocytes are consistently detected and may contribute to the overall antitumor immune activity (79).

### 3.2 TILs in shaping immunotherapy outcomes

**Figure 1:**
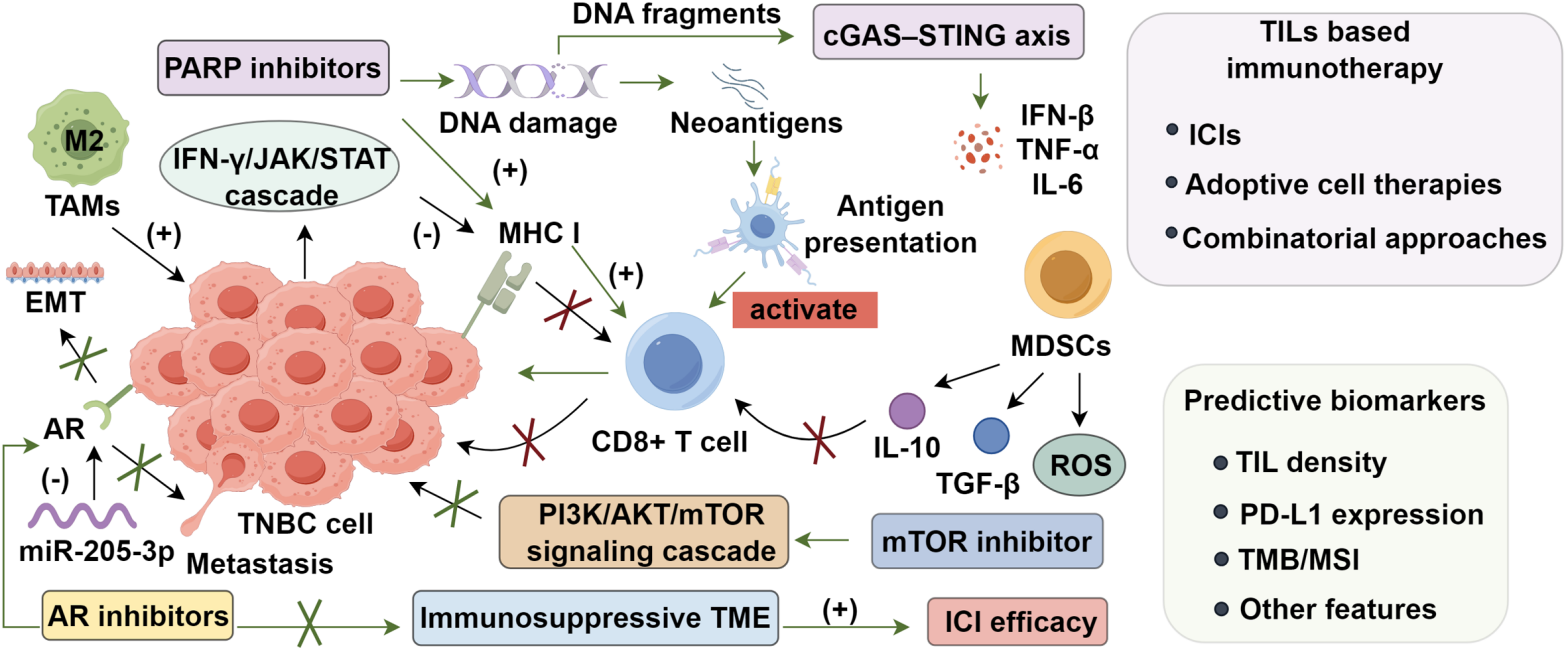
Integrated molecular signaling and immune surveillance pathways in triple-negative breast cancer.

Despite the dense infiltration of TILs and elevated inflammatory gene expression that render TNBCs highly immunogenic (80, 81), a substantial subset of patients exhibits primary or acquired resistance to immune checkpoint inhibitors (82). A key driver of primary resistance is the downregulation of major histocompatibility complex class I (MHC-I), which impairs cytotoxic T lymphocyte (CTL) recognition and facilitates immune escape (83–85), often via genetic alterations or cytokine-mediated pathways disrupting MHC-I regulation (86). Central to this regulation is the IFN-γ/JAK/STAT axis (87–90), wherein prolonged activation paradoxically suppresses MHC-I expression, diminishing CD8^+^ T cell recognition (91, 92). To counteract this, epigenetic modulators such as histone deacetylase inhibitors (HDACis) and DNA methyltransferase inhibitors (DNMTis) have shown promise in restoring MHC-I transcription (93, 94), while exogenous type I and II interferons (IFN-α/β and IFN-γ) are under clinical evaluation for enhancing antigen presentation through JAK/STAT signaling (95, 96). These strategies may synergize with ICIs to re-enable tumor immune visibility. Acquired resistance frequently involves exclusion of effector T cells from tumor parenchyma, typifying immunologically “cold” tumors shielded by stromal barriers and immunoregulatory components (97, 98). In parallel, immunosuppressive infiltrates—most notably myeloid-derived suppressor cells (MDSCs) and M2-polarized tumor-associated macrophages (TAMs)—further attenuate immune efficacy (99–102). MDSCs suppress T cell activation via reactive oxygen species (ROS) and cytokines such as IL-10 and TGF-β (103–107). Mechanistically, ROS disrupt T cell receptor (TCR) signaling, impair IL-2 production, and nitrate the CD3ζ chain, compromising cytotoxic function (108). Concurrently, IL-10 impairs antigen-presenting cell (APC) activity and promotes M2 TAM polarization (109). Additionally, MDSC-expressed arginase-1 (ARG1) depletes extracellular L-arginine, arresting T cell proliferation and inducing anergy (110). These converging suppressive circuits—spanning impaired antigen presentation, T cell exclusion, and immunosuppressive cell accumulation—jointly reinforce immune resistance. This complex immunological crosstalk within the TNBC tumor microenvironment presents a major challenge for improving ICI efficacy and underscores the need for integrative therapeutic approaches (**Figure 1**).

### 3.3 Synergistic approaches in immunotherapy combinations based on TIL

To overcome the inherent limitations of monotherapeutic approaches, increasing attention has shifted toward combinatorial immunotherapy frameworks. Among the most thoroughly validated regimens is the co-administration of ICIs alongside conventional chemotherapy (111). Cytotoxic agents can provoke immunogenic forms of tumor cell death, facilitating the release of tumor-associated antigens, which in turn activates antigen-presenting cells such as dendritic cells, triggers T-cell priming, and heightens tumor immunogenicity (112, 113). This cascade of immune activation forms the mechanistic basis for the clinical efficacy observed in trials assessing chemo-immunotherapy combinations. A landmark Phase III trial, KEYNOTE-522, illustrated that incorporating pembrolizumab with neoadjuvant chemotherapy substantially enhanced both pCR and event-free survival (EFS) in early-stage, high-risk TNBC patients. These benefits were sustained regardless of whether pembrolizumab was continued postoperatively or confined to the neoadjuvant setting, underscoring the synergistic potential of PD-1 inhibition in this context (114). Reinforcing these findings, an open-label Phase II study (NCT03639948) confirmed that combining pembrolizumab with an anthracycline-based chemotherapeutic backbone yielded superior pCR rates and extended EFS in individuals with TNBC, thereby validating the therapeutic contribution of checkpoint blockade (115).

In addition to chemotherapy, a broad array of adjunctive strategies is under active clinical exploration. Dual immune checkpoint inhibition, which has shown additive or synergistic effects in other immunogenic tumors, is currently being evaluated in TNBC cohorts (116, 117). Radiotherapy represents another complementary modality; apart from directly inducing tumor cell death, it reshapes the immune landscape by promoting cross-presentation of antigens and depleting immunosuppressive cell subsets (118, 119). Furthermore, co-targeting DNA damage repair pathways through poly (ADP-ribose) polymerase (PARP) inhibition in combination with ICIs offers a dual mechanism: destabilizing the tumor genome while concurrently enhancing immune visibility (120, 121). Emerging avenues also involve personalized neoantigen-based vaccines aimed at activating CD8^+^ T cells with tumor specificity, as well as adoptive immunotherapies including infusion of TILs and chimeric antigen receptor T (CAR-T) cells approaches designed to expand and diversify the cytotoxic immune repertoire (122–124). When integrated with checkpoint inhibitors, these interventions hold potential to overcome both intrinsic and adaptive resistance mechanisms that diminish the efficacy of monotherapies (125). Nevertheless, the enhanced efficacy achieved through combination regimens must be weighed against the increased risk of toxicity. Overlapping adverse effects, particularly immune-related toxicities (irAEs), necessitate careful dosing strategies and stringent clinical monitoring (126). Going forward, clinical trial designs should prioritize biomarker-guided patient stratification and evidence-based sequencing strategies to optimize therapeutic efficacy while maintaining drug tolerability (**Supplementary Table S1**).

### 3.4 Tumor-infiltrating lymphocytes: pivotal biomarkers for immunotherapeutic outcomes in TNBC

As immunotherapy gains prominence in TNBC, identifying reliable biomarkers to guide therapeutic stratification remains a critical priority. Although ICB has reshaped clinical paradigms, patient responses remain heterogeneous, underscoring the limitations of current predictors. Programmed death-ligand 1 (PD-L1) is widely used, yet variability in detection methods, scoring thresholds, and cellular compartments analyzed complicates standardization and cross-trial comparison (127, 128). TILs, strongly correlated with ICB benefit in immunogenic tumors, have emerged as robust prognostic markers. Integrative models combining TIL quantification and PD-L1 expression offer enhanced predictive resolution. Concurrently, tumor mutational burden (TMB) and microsatellite instability (MSI) are gaining traction as surrogate indicators of immune responsiveness (129). Elevated TMB is hypothesized to increase neoantigen load, thereby enhancing immune system visibility, while MSI-high status, previously validated in other malignancies as predictive of ICB sensitivity, may also define responsive TNBC subgroups. The immunological architecture of TNBC is increasingly understood to be modulated by emerging inhibitory receptors, including LAG-3, TIM-3, TIGIT, and VISTA, which collectively orchestrate immune evasion (130–132). These co-inhibitory molecules are frequently co-expressed with PD-1 on dysfunctional T cells, exerting suppressive effects within the TME (133, 134). Notably, LAG-3 blockade has demonstrated efficacy in melanoma, prompting investigation in breast cancer. TIM-3 and TIGIT impair CD8^+^ T cell cytotoxicity and contribute to resistance in immunologically “cold” tumors (135). The TIGIT/CD155 axis in particular has been implicated in metabolic suppression of CD8^+^ T cells via PI3K/AKT/mTOR signaling (132). VISTA, expressed on myeloid and dendritic cell subsets, exerts broad immunoregulatory effects. Its inhibition reprograms tumor-associated macrophages toward pro-inflammatory phenotypes, enhances CD8^+^ T cell infiltration, and amplifies effector responses (136). These mechanistic insights underscore the therapeutic rationale for combining next-generation checkpoint inhibitors with existing immunotherapies to overcome resistance. Optimizing TNBC treatment will require comprehensive immune profiling strategies that integrate TIL density, PD-L1 status, TMB, and emerging immunogenomic markers. A deeper understanding of immune escape pathways will inform rational design of combination regimens and accelerate precision immunotherapy. The strategic incorporation of immune modulators, such as bispecific antibodies and engineered cell therapies, into current frameworks holds substantial promise for improving clinical outcomes in TNBC (137, 138).

## 4 Conclusion

TNBC presents a major therapeutic challenge due to its aggressive biology, absence of hormone receptors, and pronounced heterogeneity. Recent molecular insights, such as DNA repair defects, PI3K/AKT/mTOR dysregulation, and androgen receptor expression, have reshaped therapeutic strategies. Targeted agents including PARP inhibitors and ADCs demonstrate efficacy in biomarker-defined subsets, while combinatorial approaches aim to overcome resistance and extend response durability. Angiogenesis modulation and intracellular pathway blockade offer additional avenues for precision therapy, contingent upon rational molecular stratification. Concurrently, TNBC’s immunogenic landscape, typified by abundant TILs and immune checkpoint expression, supports the integration of immunotherapies. However, resistance, driven by defective antigen presentation, T cell exclusion, and immunosuppressive infiltrates, necessitates combination strategies. Synergistic regimens combining immune checkpoint inhibitors with chemotherapy, radiotherapy, PARP inhibition, or emerging modulators hold potential to reshape the tumor microenvironment and augment cytotoxic immunity. Biomarker-guided immunotherapy, leveraging TIL density, PD-L1 status, TMB, and next-generation targets such as LAG-3 and TIGIT, is crucial to patient selection. Future research should emphasize rational treatment sequencing and toxicity mitigation in combination regimens. Additionally, dynamic biomarker platforms, including longitudinal immune profiling and spatial transcriptomics, may refine therapeutic timing and responsiveness. Integrative innovations such as next-generation ADCs, bispecific antibodies, and engineered cellular therapies (such as TILs and CAR-T) offer a path to consolidating targeted and immune-based modalities. Together, these evolving paradigms chart a path toward transforming TNBC into a model for immunogenomic precision oncology.
